# Gene genealogies indicates abundant gene conversions and independent evolutionary histories of the mating-type chromosomes in the evolutionary history of *Neurospora tetrasperma*

**DOI:** 10.1186/1471-2148-10-234

**Published:** 2010-07-31

**Authors:** Audrius Menkis, Carrie A Whittle, Hanna Johannesson

**Affiliations:** 1 Uppsala BioCenter, Department of Forest Mycology and Pathology, Swedish University of Agricultural Sciences, Uppsala, Sweden; 2 Department of Evolutionary Biology, Uppsala University, Uppsala, Sweden

## Abstract

**Background:**

The self-fertile filamentous ascomycete *Neurospora tetrasperma *contains a large (~7 Mbp) and young (< 6 MYA) region of suppressed recombination within its mating-type (*mat*) chromosomes. The objective of the present study is to reveal the evolutionary history, including key genomic events, associated with the various regions of the *mat *chromosomes among ten strains representing all the nine known species (lineages) contained within the *N. tetrasperma *species complex.

**Results:**

Comparative analysis of sequence divergence among alleles of 24 *mat-*linked genes (*mat A *and *mat a*) indicates that a large region of suppressed recombination exists within the *mat *chromosome for each of nine lineages of *N. tetrasperma sensu latu*. The recombinationally suppressed region varies in size and gene composition among lineages, and is flanked on both ends by normally recombining regions. Genealogical analyses among lineages reveals that eight gene conversion events have occurred between homologous *mat *A and *mat a-linked *alleles of genes located within the region of restricted recombination during the evolutionary history of *N. tetrasperma*.

**Conclusions:**

We conclude that the region of suppressed recombination in the *mat *chromosomes has likely been subjected to independent contraction and/or expansion during the evolutionary history of the *N. tetrasperma *species complex. Furthermore, we infer that gene conversion events are likely a common phenomenon within this recombinationally suppressed genomic region. We argue that gene conversions might provide an efficient mechanism of adaptive editing of functional genes, including the removal of deleterious mutations, within the young recombinationally suppressed region of the *mat *chromosomes.

## Background

*Neurospora tetrasperma *is a self-fertile filamentous ascomycete containing a large (~7 Mbp) and young (< 6 MYA) region of suppressed recombination within its mating-type (*mat*) chromosomes. The *mat *chromosomes are the largest contained within the *N. tetrasperma *genome, consisting of more than 9 Mbp and 2000 ORFs http://www.broad.mit.edu/annotation/genome/neurospora/. As in most filamentous ascomycetes, the *N. tetrasperma mat *chromosomes contain the *mat *locus, which determines haploid sexual identity and is composed of two highly dissimilar alleles (*mat A *and *mat a *idiomorphs) [1,2]. In contrast to self-incompatible (heterothallic) *Neurospora *species such as *N. crassa*, which require a partner containing nuclei of opposite mating-type for reproduction [2], *N. tetrasperma *is self-fertile (pseudohomothallic), such that heterokaryotic sexual spores contain haploid nuclei of both mating types that are required for reproduction [3,4]. Modified programs of meiosis and spore development in *N. tetrasperma *lead to the packaging of two haploid nuclei of opposite mating type into each sexual progeny, and a key feature of the *N. tetrasperma mat *chromosomes is the presence of a large recombinationally suppressed region surrounding (and including) the *mat *locus. This feature makes *N. tetrasperma *a highly suitable model system for the study of divergence in recombinationally suppressed regions, since self-fertilization renders normally recombining genes largely homoallelic while restriction of recombination leads to allelic divergence [5,6].

The *N. tetrasperma mat *chromosomes share many features with dimorphic plant and animal sex chromosomes. For example, the region of suppressed recombination has been found to contain evolutionary strata, i.e. segments with different levels of divergence among *mat A *and *mat a *linked alleles, which is consistent with expansion of the region of suppressed recombination over time [6]. Furthermore, they contain normally recombining regions, which flank the segment of suppressed recombination on both ends [6-8]; each of which are traits found in ancient sex chromosomes [6,9,10]. In contrast to most sex chromosomes, however, the *N. tetrasperma *region of suppressed recombination is relatively young, and thus has not undergone the massive degeneration observed in sex chromosomes from most other taxa, e.g. gene losses and saturated divergence levels in the Y chromosome of humans and *Drosophila *[11,12], which prevents the study of early genomic changes in sex chromosome evolution in those systems [9,13]. Thus, the *N. tetrasperma mat *chromosomes provide an effective model system to reveal the genomic events associated with early stages of sex chromosome evolution. In particular, this system is especially suited for the study of the evolutionary history of the region of recombination suppression, including genomic events such as gene conversions.

Gene conversion is the non-reciprocal transfer of information from one DNA duplex to another [14]. Empirical evidence for ectopic gene conversions between paralogous genes along non-recombining sex chromosomes has been demonstrated for the Y chromosome of humans and primates [15-17] and for the avian W chromosome [18]. In addition, indications for occasional ancestral episodes of gene conversion from × to Y have been found in several organisms including humans [15,19]. It may be postulated that gene conversions between non-recombining chromosomes could counteract the accumulation of slightly deleterious mutations, which is predicted to occur in the absence of recombination [9,20,21]. If present, this phenomenon of preventing genomic degeneration by gene conversions might be highly advantageous in young regions of recombination suppression, such as in the *N. tetrasperma mat *chromosomes, because mutations would likely convert functional genes to non-functional genes at this stage of recombination suppression. Thus, an investigation of gene conversions in *N. tetrasperma mat *chromosomes is warranted.

One highly advantageous feature of *N. tetrasperma *for the study of evolutionary history and genomic events such as gene conversions in the *mat *chromosomes is the fact that this taxon is comprised of independent and closely related species. In particular, recent comparative analyses of a genetically and geographically diverse selection of strains have revealed that *N. tetrasperma *constitutes a species complex of at least nine species [5] (with largely unresolved phylogenetic relationships). These species fulfill phylogenetic species recognition criteria and are by laboratory crossings shown to be reproductively isolated, and are referred to as *N. tetrasperma *phylogenetic lineages 1-9 [5]. In addition, all these nine species are pseudohomothallic [5]. The existence of independent phylogenetic species of *N. tetrasperma *provides a novel opportunity to examine the evolutionary history of the *mat *chromosomes because genomic events, including gene conversions and variation in the border for the region of suppressed recombination, may be detected by comparison of allelic composition of genes within and among species.

The objective of the present study is to identify key genomic events in the evolutionary history of the *mat *chromosomes within *N. tetrasperm*a. For this, we obtained DNA-sequence information from 24 genes located on each of the *mat *chromosomes (*mat A *and *mat a*) for each of the nine known lineages in the *N. tetrasperma *species complex. Genes were chosen to represent different parts of the chromosome, and comparative analyses of *mat A *and *mat a*-linked alleles of these genes were performed to identify the location of the region of suppressed recombination within the lineages, and to assess whether gene conversions and other genomic events (e.g., crossovers) play a key role in the evolution of this region among these lineages.

## Results

### Sequence divergence of the alleles located on the mat chromosomes in *N. tetrasperma *lineages

**Figure 1:**
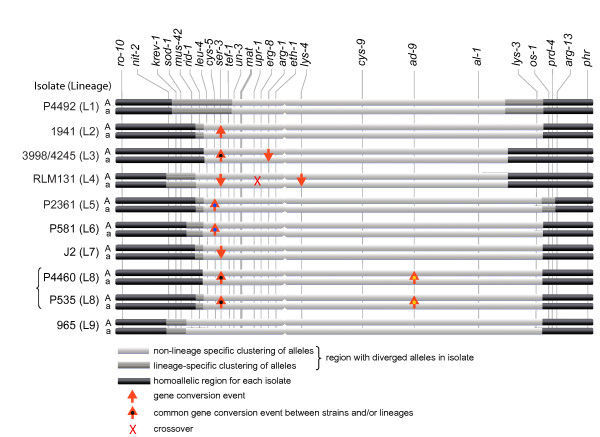
**Schematic illustration of the mating-type (*mat*) chromosomes of nine *Neurospora tetrasperma *lineages**. Approximate location of investigated genes, the *mat *locus and the centromere are indicated. Horizontal bars show the region of lineage-specific expansion of non-recombining region and instances of gene conversions and crossovers between the two chromosomes in nine lineages of *N. tetrasperma*.

For our analyses, we generated a dataset consisting of partial sequences of 24 genes located on the *mat *chromosomes in *N. tetrasperma*. The relative chromosomal location of the genes, based on gene order in *N. crassa*, is shown in Figure 1. The sequences were obtained from 20 single mating-type strains of *N. tetrasperma *(ten pairs of *mat A *and *mat a *strains originating from heterokaryotic strains isolated from nature, see Methods and Table 1). All known phylogenetic lineages of the *N. tetrasperma *species complex were represented by one pair of strains in our dataset, with the exception of lineage 8 for which two pairs were included as a reference for intraspecific variation (Table 1). The *N. tetrasperma *lineages examined in this study have been assigned alpha-numeric names ranging between L1 and L9 (Table 1). The sequence alignments are available from TreeBASE (study accession no. S10701). The gene order within the *N. tetrasperma mat *chromosomes was assumed to parallel that observed in *N. crassa *in all our analyses (based on previous findings [6,22]; see http://www.broadinstitute.org/annotation/genome/neurospora/.

**Table 1:** Fungal material of *Neurospora tetrasperma *used in the study

**Wild-type strains of *N. tetrasperma***^**1**^				Mating type	Geographic origin
		
**Strain ID**^**2**^	Phylogenetic **Lineage**^**3**^	**Heterokaryon**^**4**^	**Homokaryon**^**5**^		
L1A	1	P4492	FGSC 9033	*A*	Franklin, Louisiana
L1a			FGSC 9034	*a*	
L2A	2	FGSC 1941	1941A^6^	*A*	La Belle, Florida
L2a			1941a^6^	*a*	
L3A	3	unknown	FGSC 3998^7^	*A*	Raleigh, North Carolina
L3a		unknown	FGSC 4245^7^	*a*	
L4A	4	RLM131	FGSC 7585	*A*	Coba, Mexico
L4a			FGSC 7586	*a*	
L5A	5	P2361	P4371	*A*	Ahipara, New Zealand
L5a			P4372	*a*	
L6A	6	P581	FGSC 2508	*A*	Lihue, Hawaii
L6a			FGSC 2509	*a*	
L7A	7	J2	FGSC 9045	*A*	Franklin, Louisiana
L7a			FGSC 9046	*a*	
L8(1)A	8	P4460	FGSC 9030	*A*	Franklin, Louisiana
L8(1)a			FGSC 9031	*a*	
L8(2)A		P535	535A^6^	*A*	Perkins, Louisiana
L8(2)a			535a^6^	*a*	
L9A	9	FGSC 965	965A^6^	*A*	Liberia
L9a			965a^6^	*a*	

^1 ^FGSC = The Fungal Genetics Stock Center. P = accession number from the Perkins collection of *Neurospora *curated by FGSC. RLM = strain originally collected by R. L. Metzenberg. J = strain originally collected by D. Jacobson

^2 ^Strain identification used in this study

^3 ^Genetically and reproductively isolated species described in [5]

^4 ^Heterokaryotic (n+n) strain of *N. tetrasperma *originally isolated from nature

^5 ^Homokaryons are haploid (n), single mating-type, components derived from the heterokaryon

^6 ^Homokaryons segregated by single ascospore isolation in [5]

^7 ^The corresponding opposite mating-type homokaryon of the same heterokaryon was not available from the culture collection and the originating heterokaryon was either unknown or unavailable.

The divergence level (*d*_N_, *d*_S _and intron polymorphisms) between the *mat A *and *mat a*-linked alleles for each of the ten paired comparisons is shown in Table 2. The data shows that the alleles of nearly all genes located in the two chromosome flanks were homoallelic for each pair (Table 2). We interpret the homoallelism as a consequence of recombination; this is because genes in a normally recombining chromosomal segment should be identical or nearly identical between the two *mat *chromosomes in a highly self-fertilizing taxon such as *N. tetrasperma *[5]. Three exceptions to this pattern were found for the chromosome pairs: L3A-L3a shows divergence for genes *os-1 *and *phr*, L4A-L4a for the genes *nit-2 *and *krev-1*, and L5A-L5a for the gene *prd-4 *(Table 2). In each of these cases the divergence was caused by a single nucleotide polymorphism, and could result from very rare outcrossing events in this highly inbred taxon [5]. Altogether, these results demonstrate that genes are highly homoallelic in the chromosome flanks of the *mat *chromosomes of natural strains of *N. tetrasperma*, and that this is most likely due to normal recombination.

**Table 2:** Allele divergence between pairs of single mating-type component strains belonging to nine species of the *Neurospora tetrasperma *species complex

**Locus**^**a**^	Strain **pair**^**b**^	L1A-L1a			L2A-L2a			L3A-L3a			L4A-L4a			L5A-L5a			L6A-L6a			L7A-L7a			L8(1)A-L8(1)a			L8(2)A-L8(2)a			L9A-L9a		
	**Lineage**^**c**^	1			2			3			4			5			6			7			8			8			9		
	**Exon** **sequences (bp)**	***d***_**N**_	***d***_**S**_	**i**^**d**^	***d***_**N**_	***d***_**S**_	**i**^**d**^	***d***_**N**_	***d***_**S**_	**i**^**d**^	***d***_**N**_	***d***_**S**_	**i**^**d**^	***d***_**N**_	***d***_**S**_	**i**^**d**^	***d***_**N**_	***d***_**S**_	**i**^**d**^	***d***_**N**_	***d***_**S**_	**i**^**d**^	***d***_**N**_	***d***_**S**_	**i**^**d**^	***d***_**N**_	***d***_**S**_	**i**^**d**^	***d***_**N**_	***d***_**S**_	**i**^**d**^

*ro-10*	590	0	0	0	0	0	0	0	0	0	0	0	0	0	0	0	0	0	0	0	0	0	0	0	0	0	0	0	0	0	0
*nit-2*	3293	0	0	0	0	0	0	0	0	0	0	0.001	0	0	0	0	0	0	0	0	0	0	0	0	0	0	0	0	0.002	0	0
*krev-1*	521	0	0.008	11	0	0	0	0	0	0	0	0	1	0	0	0	0	0	0	0	0	0	0	0	0	0	0	0	0	0.025	0
*sod-1*	465	0	0.017	3	0	0	0	0	0	0	0	0	0	0	0	0	0	0	0	0	0	0	0	0	0	0	0	0	0	0.009	0
*mus-42*	1680	0	0.003	-	0	0	-	0	0	-	0	0	-	0	0	-	0.001	0.018	-	0	0	-	0	0	-	0	0	-	0.006	0.060	-
*rid*	2560	0	0.005	-	0.001	0.004	-	0	0	-	0.010	0.033	-	0.001	0	-	0	0	-	0.001	0	-	0	0	-	0.001	0	-	0.031	0.147	-
*leu-4*	1863	0	0	0	0.002	0.028	9	0	0.028	3	0.001	0.007	5	0.001	0.028	9	0.001	0.028	10	0	0.030	7	0	0.014	4	0	0.014	4	0.004	0.059	8
*cys-5*	883	0	0	-	0	0.024	-	0.005	0.024	-	0.002	0.034	-	0	0	-	0	0	-	0.002	0.029	-	0.003	0.015	-	0.003	0.020	-	0.003	0.070	-
*ser-3*	922	0.004	0.025	-	0	0	-	0	0	-	0.002	0.004	-	0.007	0.047	-	0.007	0.047	-	0.002	0.004	-	0	0	-	0.002	0	-	0.006	0.074	-
*tef-1*	476	0	0	3	0	0.038	18	0	0.048	17	0	0.019	23	0	0.019	16	0	0.029	16	0	0.009	13	0	0.019	19	0	0.019	20	0	0.048	23
*un-3*	1924	0.001	0.031	-	0.002	0.048	-	0.002	0.036	-	0.002	0.028	-	0.002	0.048	-	0.003	0.048	-	0	0.048	-	0.001	0.019	-	0.001	0.019	-	0.002	0.045	-
*mat*																															
*upr-1*	2888	0.017	0.053	-	0.021	0.057	-	0.016	0.065	-	0.016	0.048	-	0.021	0.055	-	0.021	0.055	-	0.015	0.054	-	0.004	0.012	-	0.004	0.012	-	0.031	0.079	-
*erg-8*	1429	0	0.037	3	0.005	0.063	6	0	0.069	4	0.001	0.066	4	0.004	0.063	6	0.003	0.059	6	0.001	0.046	5	0.001	0.037	3	0.001	0.037	3	0.003	0.076	8
*arg-1*	1119	0	0.008	10	0.001	0.043	13	0	0	0	0	0.043	11	0.001	0.051	13	0.001	0.047	13	0	0.031	8	0	0.019	10	0	0.019	10	0	0.051	19
*eth-1*	866	0.005	0.005	3	0.002	0.029	5	0.003	0.029	3	0.002	0.034	3	0.002	0.029	5	0.002	0.029	5	0	0.034	4	0.002	0.024	2	0.002	0.024	2	0.002	0.054	5
centromere																															
*lys-4*	1055	0.001	0.049	23	0.004	0.053	30	0.005	0.057	9	0	0	0	0.004	0.061	17	0.004	0.061	17	0.006	0.038	7	0.004	0.024	13	0.020	0.020	0	0.006	0.076	15
*cys-9*	907	0	0.047	7	0	0.082	8	0	0.037	7	0	0.042	6	0	0.082	8	0	0.082	8	0.002	0.023	3	0	0.037	5	0	0.037	9	0	0.057	14
*ad-9*	609	0.004	0.035	2	0.002	0.072	1	0.002	0.057	5	0.004	0.064	3	0.022	0.072	1	0.002	0.072	1	0.007	0.072	8	0	0.007	0	0	0.007	0	0.004	0.049	3
*al-1*	1637	0.002	0.032	1	0.004	0.073	17	0.002	0.065	18	0.001	0.029	12	0.004	0.076	17	0.005	0.073	17	0.002	0.073	1	0.005	0.053	15	0.005	0.051	15	0.004	0.054	5
*lys-3*	3324	0	0.001	0	0.001	0.062	0	0	0	0	0	0	0	0.001	0.062	0	0.001	0.062	0	0.002	0.031	1	0.001	0	0	0.001	0	0	0.002	0.052	5
*os-1*	1749	0	0	0	0	0	0	0	0.003	0	0	0	0	0	0	0	0	0	0	0	0	0	0	0	0	0	0	0	0	0	0
*prd-4*	1475	0	0	0	0	0	0	0	0	0	0	0	0	0.001	0	0	0	0	0	0	0	0	0	0	0	0	0	0	0	0	0
*arg-13*	999	0	0	0	0	0	0	0	0	0	0	0	0	0	0	0	0	0	0	0	0	0	0	0	0	0	0	0	0	0	0
*phr*	1769	0	0	0	0	0	0	0.001	0	0	0	0	0	0	0	0	0	0	0	0	0	0	0	0	0	0	0	0	0	0	0

^a ^Genes are listed from the left to the right flank of the mating-type chromosome according to gene order in *N. crassa*. The location of the *mat*-locus and the centromere is indicated.

^b ^Full strain ID can be found in Table 1. All pairs, except L3A-L3a, originates from one wild-type heterokaryon of *N. tetrasperma*

^c ^Genetically and reproductively isolated species described in [5].

^d ^Number of single nucleotide polymorphisms in intron sequences of the genes

In contrast to the chromosome flanks, the results show that the vast majority of the genes located in the central segment of the *mat *chromosomes have marked divergence between the *mat A *and *mat a*-linked alleles (Table 2). We attribute this result to suppressed recombination in this segment of the chromosomes for all lineages of *N. tetrasperma*. However, lineage-specific exceptions to this pattern were also found, for example the gene *lys-4 *showed divergence among *mat A *and *mat a*-linked alleles in all lineages except for lineage 4, in which this gene was homoallelic. Based on the gene order in *N. crassa*, this gene is located in the recombinationally suppressed region of the *N. tetrasperma mat *chromosomes (Table 2).

### Genealogies of genes along the mat chromosomes of *N. tetrasperma*

**Figure 2:**
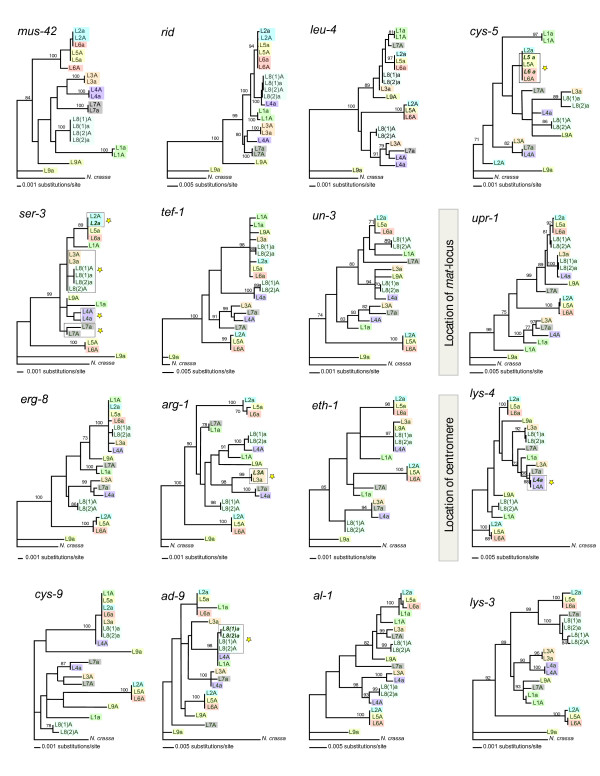
**Gene genealogies of 16 genes in the region of suppressed recombination of the mating-type (*mat*) chromosomes of *Neurospora tetrasperma***. The genes are visualized horizontally in a consecutive order according to gene order in *Neurospora crassa*, from left to right flank of the chromosomes. The position of the *mat *locus and the centromere are indicated. Alleles from *mat *chromosomes originating from wild-type heterokaryons are indicated by strain ID given in Table 1, and strains of the same phylogenetic species in the *N. tetrasperma *species complex are marked with the same color. Gene conversion events are boxed and indicated by yellow stars. Alleles of genes marked in bold italic were used as template for gene conversion.

Gene genealogies of the 24 investigated genes, spanning each of *N. tetrasperma *lineages examined herein, are depicted in Figure 2 and in Additional file 1: Figure S1. Genealogies are depicted in the sequential order of the genes in *N. crassa*. For the genes located in the recombining chromosomal ends (*ro-10*, *nit-2*, *krev-1 *and *sod-1 *on the left flank and *os-1*, *prd-4*, *arg-13 *and *phr *on the right flank), alleles linked to the *mat A *and *mat a *chromosomes do not cluster separately by significantly supported branches for the various lineages, rather they cluster primarily by lineage (Additional file 1: Figure S1). In contrast, for the genes in the central part of the *mat *chromosomes, ranging from *mus-42 *to *lys-3*, the *mat A *and *mat a*-linked alleles cluster separately for at least one of the ten *N. tetrasperma *pairs examined (Figure 2). The pattern of clustering of the alleles is summarized in Figure 1.

In regions of the *mat *chromosome where recombination cessation predates divergence of the *N. tetrasperma *lineages [6], all alleles from *mat A *strains are expected to cluster separately from the alleles of the *mat a *strains in the gene genealogies. This pattern was not found in any of the genealogies (Figure 1), which could be the result of gene conversions, occasional crossovers, introgressions and/or independent origin of the recombination suppression in different lineages of *N. tetrasperma*.

### Indications of gene conversions between mat chromosomes in *N. tetrasperma*

We visually inspected windows of three neighboring genealogies along the chromosomes (Figure 1), and interpreted a gene conversion event to have taken place when two alleles of a pair (i.e., *mat A *versus *mat a*-linked alleles originating from a single lineage) cluster together in one genealogy, flanked by two genealogies in which the alleles for this specific pair are found in two different clades, at least one of which significantly supported by PAUP bootstrap analyses. For example, the two different alleles of *lys-4 *gene of L4A and L4a cluster together, while in *eth-1 *alleles from this pair cluster separately, similarly as found in the centromere-distal neighboring gene *cys-9*: this pattern was interpreted as the result of a gene conversion between the two *lys-4 *alleles of L4A and L4a, with the *mat a*-linked allele used as a template for gene conversion. Using this approach to identify potential gene conversions, a total of eight gene conversion events were found between homologous alleles of the chromosomal region of suppressed recombination (indicated by stars in Figure 2, and summarized in Figure 1).

We traced the ancestry of the aforementioned gene conversions. For this, we identified the alleles subjected to gene conversion that were identical among multiple lineages; in these cases we concluded that the gene conversion occurred before the split of the lineages. We found that one gene conversion (in *ser-3*) was traced back to a common ancestor of *N. tetrasperma *lineages 3 and 8, and one (*cys-5*) to a common ancestor of *N. tetrasperma *lineages 5 and 6. In *arg-1*, a gene conversion is assumed to have taken place early after the radiation of the *N. tetrasperma *phylogenetic lineage 3, affecting more than one heterokaryon of this lineage (L3A and L3a do not originate from the same natural heterokaryon, see Table 1). In four genes, *cys-5*, *ser-3*, *lys-4 *and *ad-9*, the clustering of the alleles in the genealogies suggests that the *mat a*-linked alleles were used as template for gene conversion, and in two genes, *ser-3 *and *arg-1*, our data suggests gene conversions in the opposite direction, i.e. the *mat A*-linked alleles were used as template (Figure 1, Figure 2).

### Evidence of occasional crossovers between the mat chromosomes in *N. tetrasperma*

The study of the gene genealogies (Figure 2) gives indications for a crossover between the *mat *chromosomes in phylogenetic lineage 4, at a location between *upr-1 *and *erg-8*. For the genes left of *erg-8 *the alleles of L4a clusters consistently together with alleles of strains L3a, L8(1)a and L8(2)a while the alleles for L4A clusters with alleles of strains L7a and L3A. In contrast, for *erg-1 *and the genes right of this gene, the alleles of L4A and L4a have swapped position in the genealogies (Figure 2). For several additional pairs of *mat *chromosomes we found other, less clear, indications of crossovers in that alleles from one mating-type component strain clustered together with alleles from sister-lineages of the opposite mating-type component (Figure 2).

### Independent histories of the mat chromosomes in *N. tetrasperma *lineages

The clustering of alleles of the genes located at the edges between the central and distal chromosomal region (i.e. *mus-42 *and *rid *on the left flank and *al-1 *and *lys-3 *on the right flank; Figure 2), suggests a lineage-specific evolutionary history in these regions of the *mat *chromosomes. For example, the *rid *genealogy shows supported divergence of the *mat A *and *mat a*-linked alleles in phylogenetic lineage 4 (L4). This is in contrast to several other lineages, which are homoallelic for *rid *and all genes left of this gene (Figure 1, 2 and Additional file 1: Figure S1). Thus, this observation indicates that *rid *is located in the region of suppressed recombination in L4, but is located in the recombining region in other lineages. A similar pattern is found for the genes on the right flanking region of the *mat *chromosomes, e.g. in gene *lys-3*, alleles of lineage 2 (L2), lineage 5 (L5) and lineage 6 (L6) are separated by significantly supported branches, while alleles of lineage 4 (L4) are identical. In totality, these data support the independent evolution of the border between the recombining and the recombinationally suppressed region among lineages in the *N. tetrasperma *species complex.

## Discussion

The present findings have shown that all known lineages in the *N. tetrasperma *species complex contain similar key features within the *mat *chromosomes. Specifically, our data have revealed firstly, that the central segment of the *mat *chromosomes contains genes wherein alleles have markedly diverged, and secondly, that this region is flanked by regions with very low, or no, divergence. Although this pattern was previously shown for the *mat *chromosomes for a single lineage (strain P581; belonging to phylogenetic lineage 6) [6], our present results demonstrate that this is a general trait observed for all lineages of *N. tetrasperma*. Based on the fact that normal recombination would be expected to homogenize the gene alleles located on the two *mat *chromosomes in a highly self-fertilizing taxon such as *N. tetrasperma *[5,6], we conclude that the present findings are best explained by the presence of suppressed recombination in the central part of the *mat *chromosomes and normal recombination within the flanking regions.

### Differences in evolutionary history of the mat chromosomes among lineages

Our data suggests that the evolutionary history of the *mat *chromosomes differs among the various lineages of *N. tetrasperma*. The phylogenetic relationship between the lineages of the *N. tetrasperma *species complex is unresolved [5], but assuming that lineage 9 is the most basal lineage of *N. tetrasperma*, one explanation for this finding is a contraction in the size of the recombinationally suppressed region in lineages 1-8 following their divergence from lineage 9. Another plausible explanation for the independent evolution is that the segment with suppressed recombination was initially a small region, which has expanded independently and to a different extent within lineages. Such "evolutionary strata", arising from successive expansion of recombination suppression, have been observed in a wide range of sex chromosomes including those from humans, chickens and plants [23-25] as well as for the *mat *chromosomes for *N. tetrasperma *phylogenetic lineage 6 (strain P581) [6]. This process is believed to be driven by natural selection for linkage between the current region of suppressed recombination and sexually antagonistic loci (i.e. beneficial to one sex but detrimental to the other) [13,26,27]. However, given that differentiated sexes do not exist in *N. tetrasperma; *individual haploid strains of either mating-type are able to produce both male and female reproductive structures, it is not known whether such sexual antagonistic processes could play a role in the emergence of evolutionary strata in this taxon.

### Gene conversions

Data from our gene genealogies indicates that gene conversions between homologous genes of the *mat *chromosomes have occurred multiple times during the evolutionary history of *N. tetrasperma*, despite the suppression of recombination. For the majority of the gene conversion events observed in this region, two homologous gene copies of the *mat *chromosomes were completely homogenized, indicating that gene conversion tracts (the DNA region affected by conversion) were large and continuous, and likely extended beyond the region of the gene investigated herein. Notably, for *ser-3*, the alleles were not completely homogenized; however given that only one or two bp differences were found among the alleles, it is not possible to differentiate between partial and complete gene conversions for this specific gene. Although tracts of gene conversion are usually relatively short in many systems, previous reports from the fungal kingdom have shown tracts of gene conversion in the same order of magnitude (1.5 kb) as the regions studied here [28].

The totality of our findings suggests that gene conversion between homologous alleles of diverging chromosomes could be a significant evolutionary force shaping young regions of suppressed recombination in the *N. tetrasperma mat *chromosomes. The rate of homogenization of individual sites of each gene by gene conversion can be estimated using the equation r=K/(2T), commonly used for estimating the accumulation of substitutions between pairs of taxa over time [29], where K is the number of gene conversion events divided by number of lineages, and T is the time since divergence of *N. tetrasperma *lineages from a common ancestor. Assuming a divergence time of *N. tetrasperma *lineages from a common ancestor of 4.6 MYA [6], the rate of homogenization for each individual site by gene conversion for the genes *cys-5*, *arg-1*, *lys-4 *and *ad-9 *is 0.012 per million years and for *ser-3 *0.048 per million years, which is one order of magnitude higher than the rate of substitutions for coding sequences (0.0037 per site and million years [30]). Accordingly, we postulate that frequent gene conversions could arise as a mechanism to counteract the effects of deleterious mutations that are predicted to arise in the young recombinationally suppressed region of the *mat *chromosomes.

In addition to *N. tetrasperma*, the role of gene conversions and/or recombination in the evolutionary history of mating-type chromosomes has been reported for other fungal systems. For example, it has been found that the evolution of the mating-type locus of *Cryptococcus neoformans *likely started by the acquisition of sex-determining genes into the mating-type region [31], a process followed by gene conversion and/or recombination giving rise to a region of suppressed recombination harboring genes of common function, e.g. pheromone production and sensing, surrounding the mating-type locus [31-33]. The pheromone genes of *C. neoformans *have been found to be arranged in a palindromic fashion, and have been subjected to gene conversion events similar to the genes involved in spermatogenesis on the human Y chromosome [10]. These findings further collaborate our conclusion that gene conversions are a common phenomenon associated with recombination suppression in the fungal mating-type loci and mating-type chromosomes.

Several mechanisms could be responsible for the onset of gene conversions in the *N. tetrasperma mat *chromosomes. Specifically, given that the *mat *chromosomes in *N. tetrasperma *are located in separate haploid nuclei during vegetative growth, we can infer that the observed gene conversions must have taken place either during sexual reproduction (meiosis) or through the process of parasexuality, i.e. a process of recombination of hereditary determinants outside of the sexual cycle first reported from the fungal kingdom by Pontecorvo [34]. Since the gene conversions were detected in the recombinationally suppressed region of the *mat *chromosomes of *N. tetrasperma*, it is plausible that the same genetic mechanism as for ectopic gene conversion could be important, as this particular process does not necessarily require the formation of the synaptonemal complex accompanied by reciprocal exchange [35,36].

### Noteworthy issues

It should be noted that an altered gene order in the different lineages of *N. tetrasperma*, due to gene translocation or multiple inversions, could result in deviations in divergence for specific genes. Gene order has previously been found to be highly conserved between *N. tetrasperma *and *N. crassa *[22] justifying our general assumption on conserved gene order between the taxa. Furthermore, in a previous study we specifically confirmed the gene order for the region comprising *leu-4*, *cys-5 and ser-3 *in strain P581 (phylogenetic lineage 6) [6], and recently acquired large scale genomic data confirms gene order for *cys-5*, *ser-3 *and *tef-1 *in strain RLM131 (phylogenetic lineage 4, unpublished data). These confirmations of a conserved gene order between *N. tetrasperma *and *N. crassa *make it possible to rule out translocation to a recombining chromosomal region as the cause of the homoallelism for specific genes such as *cys-5 *in L6A and L6a and *ser-3 *in L4A and L4a; and thus further supports the conclusion that gene conversion events are responsible for the observed homoallelism in these two cases. In the future, high coverage genomic and population data will be needed in order to better understand the full extent to which gene conversions contribute to the evolutionary history of *N. tetrasperma*.

## Conclusions

Our present analyses have revealed several fundamental factors associated with the evolution of *N. tetrasperma mat *chromosomes. In particular, our data shows that the *mat *chromosomes from the various *N. tetrasperma *lineages evolve independently, with certain lineages having different demarcation points between the recombining and the recombinationally suppressed regions as compared to other lineages. This finding is remarkable given that these lineages are very closely related and are not perfectly reproductively isolated [5]. In addition, the present data has also shown that the *mat *chromosomes contain a young region of recombination suppression, flanked by normally recombining regions, in each of the nine lineages of *N. tetrasperma*. These features are consistent with those reported for ancient sex chromosomes from animals [11,12,37-39]. Furthermore, the data demonstrates that recombination suppression is not perfectly maintained in the *N. tetrasperma mat *chromosomes; this is based on the fact that gene genealogies show that gene conversions and occasional crossovers have arisen in the recombinationally suppressed region within this taxonomic group. We conclude that gene conversions could be a common phenomenon, which counteracts the deleterious effects of mutations during early stages of recombination suppression in *N. tetrasperma mat *chromosomes. Overall, the present data demonstrate the high utility of this fungal model system in revealing traits associated with early stages of recombination suppression. Furthermore, the findings described herein provide a framework for further analysis of how the *mat *chromosomes evolve within and among *N. tetrasperma *lineages, which will be key for the usage of this taxonomic group as a model system for the study of early stages of recombination suppression in sex chromosomes.

## Methods

*N. tetrasperma *is a self-fertile taxon. Specifically, sexual spores and vegetative cells in this taxon contain haploid nuclei of both mating types (*mat A *and *mat a*), resulting in heterokaryotic individuals capable of completing the life-cycle on their own; a mating-system called pseudohomothallism [3,4]. Pseudohomothallism is believed to have evolved in *N. tetrasperma *less than 6 MYA and is not present in other, closely related, *Neurospora *species which are self-incompatible (heterothallic) and require mating between haploid individuals of opposite mating types, *mat A *and *mat a *[3,4,6,40-42]. Nonetheless, it has been found that heterokaryotic *N. tetrasperma *strains (containing both *mat A *and *mat a *nuclei) can be separated into two homokaryotic (i.e. haploid), single mating-type component strains [3,43], which allows the study of the divergence among gene alleles located on the *mat A *and *mat a *chromosomes in a single strain [6-8].

### Fungal strains

Ten pairs of homokaryotic, single mating-type component, strains of *Neurospora tetrasperma *were used in this study. All except one pair (FGSC3998 and FGSC4245: phylogenetic lineage 3) originates from single wild-type heterokaryons. The material represents the nine phylogenetically and biologically recognized species of *N. tetrasperma*, which are referred to as *N. tetrasperma *phylogenetic lineages 1-9 until the formal nomenclature is settled [5]. From each lineage of *N. tetrasperma*, one pair of single mating-type component strains were included, except for lineage 8 from which we included two pairs of strains. The strains were obtained from the Fungal Genetics Stock Center (FGSC), University of Missouri. Strain names, origin and lineage affinity are given in Table 1, and additional information about the strains can be found in [5].

### DNA manipulations

DNA from the 20 single mating-type component strains was extracted for a previous study [5]. For the present study, partial or complete sequences of 24 genes (on average 1460 bp coding sequences/gene) located on the *mat *chromosomes, were PCR amplified and sequenced from all 20 strains. Description of the loci, specific primers for their amplification, PCR conditions, procedures for DNA sequencing and software used for sequence analyses can be found in [6].

### Evolutionary genetic analyses

Synonymous and non-synonymous nucleotide divergence rates of homologous alleles (*d*_S _and *d*_N_, respectively) were estimated for the alleles of the *mat *chromosomes of heterokaryotic strains, by using DNAsp version 4.10.9 [44].

### Phylogenetic analyses

Sequences were aligned for each gene using the Clustal W algorithm of BioEdit version 7.0.5.2 [45]. Gene trees were derived by maximum likelihood (ML) analyses using heuristic searches and the tree bisection-reconnection (TBR) branch-swapping algorithm in PAUP 4.0b10 [46]. Statistical support for phylogenetic grouping was assessed by bootstrap and Bayesian posterior probability analyses. For bootstrap analyses in PAUP 4.0b10, we used 1000 replicate datasets with the random addition of sequences during each heuristic search, and a threshold for significance of bootstrap branch support values of ≥70%, which have been shown likely to correspond to confidence levels of 95% [47]. Bayesian posterior probabilities were calculated for the clades of the trees using MrBayes 3.1.2. [48], as described previously [5]. The threshold for significant Bayesian branch support was set to 0.95 [49].

## Authors' contributions

AM carried out the molecular work, performed the phylogenetic analyses and drafted the manuscript. CAW interpreted the data and drafted the manuscript. HJ conceived of the study, participated in its design and coordination, and drafted the manuscript. All authors read and approved the final manuscript.

## Supplementary Material

Additional file 1 **Gene genealogies of 8 genes in the two chromosomal flanks of the *mat *chromosomes of *Neurospora tetrasperma*.** Alleles from homokaryotic single mating-type component originating from wild-type heterokaryons were marked with the same color. Gene genealogies of 8 genes in the two chromosomal flanks of the *mat *chromosomes of *Neurospora tetrasperma*. Click here for file
