# Supplementary material for: Gene genealogies indicates abundant gene conversions and independent evolutionary histories of the mating-type chromosomes in the evolutionary history of Neurospora tetrasperma
Source: BMC Evol Biol. 2010 Jul 31;10:234. doi: 10.1186/1471-2148-10-234 (PMC2923516; doi:10.1186/1471-2148-10-234)

Genealogies of genes located in the recombining left flank of the mat chromosomes of *N. tetrasperma*

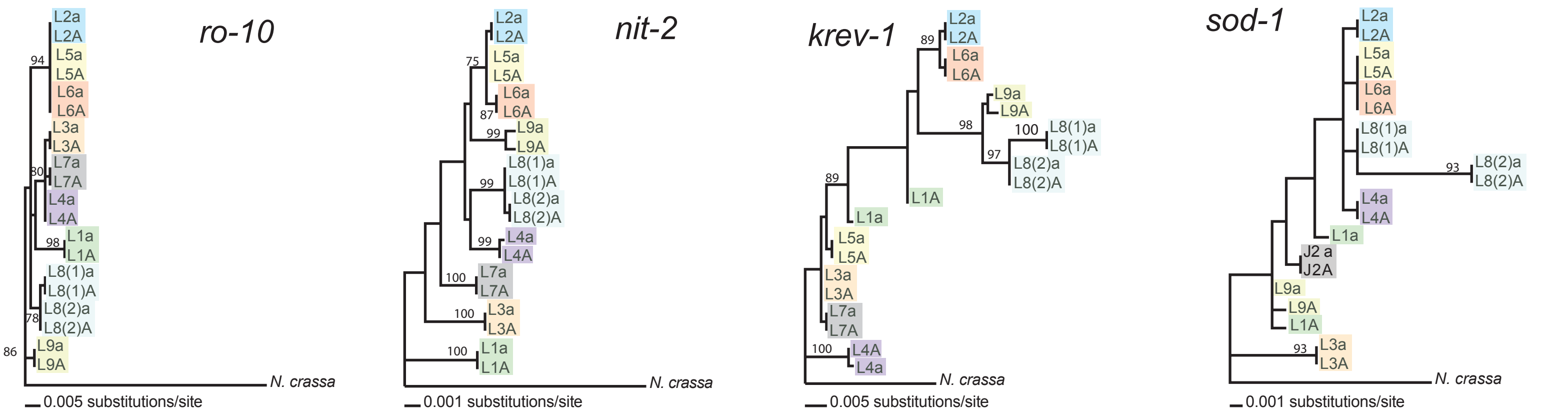

Genealogies of genes located in the recombining right flank of the *mat* chromosome of *N. tetrasperma*

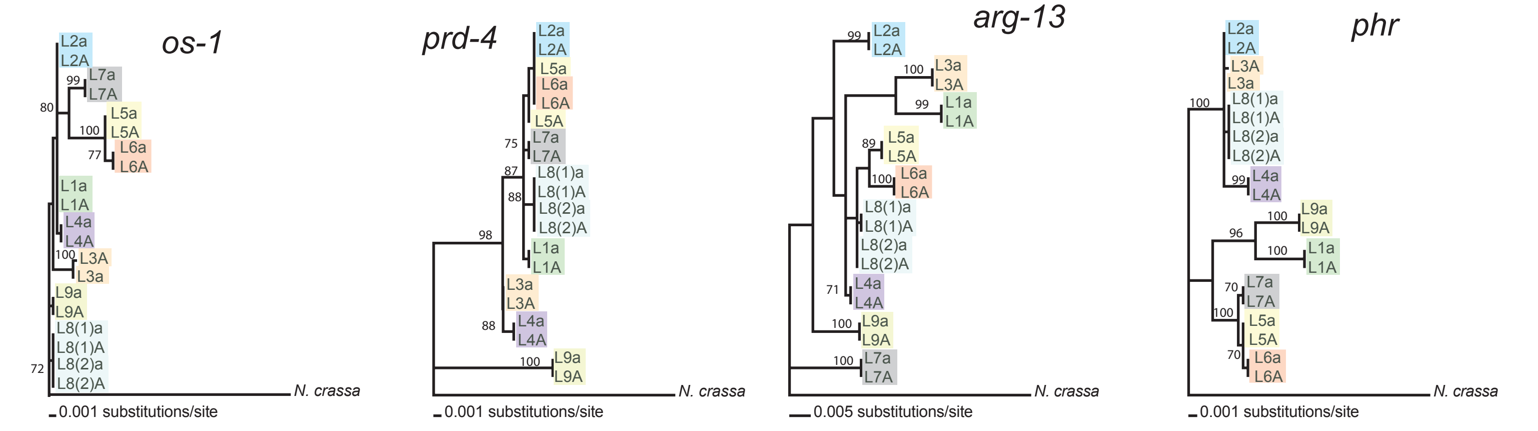

Supplement: Additional file 1 — Gene genealogies of 8 genes in the two chromosomal flanks of the mat chromosomes of Neurospora tetrasperma. Alleles from homokaryotic single mating-type component originating from wild-type heterokaryons were marked with the same color. Gene genealogies of 8 genes in the two chromosomal flanks of the mat chromosomes of Neurospora tetrasperma. [file 1471-2148-10-234-S1.PDF]
